# Maternal diet quality and associations with plasma lipid profiles and pregnancy-related cardiometabolic health

**DOI:** 10.1007/s00394-023-03244-3

**Published:** 2023-08-30

**Authors:** Paige F. van der Pligt, Konsita Kuswara, Sarah A. McNaughton, Gavin Abbott, Sheikh Mohammed Shariful Islam, Kevin Huynh, Peter J. Meikle, Aya Mousa, Stacey J. Ellery

**Affiliations:** 1 Institute for Physical Activity and Nutrition (IPAN), School of Exercise and Nutrition Sciences, Deakin University, Geelong, 3220 Australia; 2 Department of Nutrition and Dietetics, Western Health, Footscray, Australia; 3 School of Exercise and Nutrition Sciences, Deakin University, Geelong, VIC 3220 Australia; 4 Baker Heart and Diabetes Institute, 75 Commercial Road, Melbourne, VIC 3004 Australia; 5 Faculty of Medicine, Nursing and Health Sciences, Monash University, Melbourne, Australia; 6 Monash Centre for Health Research and Implementation (MCHRI), School of Public Health and Preventive Medicine, Monash University, Clayton, VIC 3168 Australia; 7 The Ritchie Centre, Hudson Institute of Medical Research, Clayton, VIC Australia; 8 Department of Obstetrics and Gynaecology, Monash University, Clayton, VIC Australia

**Keywords:** Diet quality, Pregnancy, Cardiovascular disease, Cardiometabolic disease, Lipidomics

## Abstract

**Purpose:**

To assess the relationship of early pregnancy maternal diet quality (DQ) with maternal plasma lipids and indicators of cardiometabolic health, including blood pressure (BP), gestational diabetes mellitus (GDM) and gestational weight gain (GWG).

**Methods:**

Women (*n* = 215) aged 18–40 years with singleton pregnancies were recruited at 10–20 weeks gestation. Diet quality was assessed by the Dietary Guideline Index, calculated at early ([mean ± SD]) (15 ± 3 weeks) and late (35 ± 2 weeks) pregnancy. Lipidomic analysis was performed, and 698 species across 37 lipid classes were measured from plasma blood samples collected at early (15 ± 3 weeks) and mid (27 ± 3 weeks)-pregnancy. Clinical measures (BP, GDM diagnosis, weight) and blood samples were collected across pregnancy. Multiple linear and logistic regression models assessed associations of early pregnancy DQ with plasma lipids at early and mid-pregnancy, BP at three antenatal visits, GDM diagnosis and total GWG.

**Results:**

Maternal DQ scores ([mean ± SD]) decreased significantly from early (70.7 ± 11.4) to late pregnancy (66.5 ± 12.6) (*p* < 0.0005). At a false discovery rate of 0.2, early pregnancy DQ was significantly associated with 13 plasma lipids at mid-pregnancy, including negative associations with six triglycerides (TGs); TG(54:0)[NL-18:0] (neutral loss), TG(50:1)[NL-14:0], TG(48:0)[NL-18:0], TG(52:1)[NL-18:0], TG(54:1)[NL-18:1], TG(50:0)[NL-18:0]. No statistically significant associations were found between early pregnancy DQ and BP, GDM or GWG.

**Conclusion:**

Maternal diet did not adhere to Australian Dietary Guidelines. Diet quality was inversely associated with multiple plasma TGs. This study provides novel insights into the relationship between DQ, lipid biomarkers and cardiometabolic health during pregnancy.

**Supplementary Information:**

The online version contains supplementary material available at 10.1007/s00394-023-03244-3.

## Introduction

Pregnancy is a critical life stage whereby maternal nutrition significantly impacts the immediate and long-term health of the mother and offspring [1]. Globally, unhealthy diets, which are energy dense but lack nutritional quality, comprise a significant behavioural risk factor for the development of chronic disease [2]. Sub-optimal pregnancy nutrition has been linked with increased inter-generational risk factors for cardiometabolic disease, including elevated blood lipids, hypertension, impaired glucose tolerance, and long-term obesity [2–4].

Pregnancy has been regarded as a ‘stress test’ for development of cardiometabolic conditions including cardiovascular disease (CVD) [5], a major cause of morbidity and mortality globally [6]. Physiological adaptations that occur with pregnancy progression, including metabolic and haemodynamic changes, increase stress on the maternal cardiovascular system [7]. In addition, common pregnancy complications including preeclampsia and gestational diabetes mellitus (GDM) often driven by obesity and excess gestational weight gain (GWG), determined by pre-pregnancy BMI [7, 8], significantly increase the risk of postpartum maternal CVD and cardiometabolic disease [8, 9]. Roughly two-thirds of all women gain more weight than recommended during pregnancy [10]. Hypertensive disorders of pregnancy affect 10% of pregnancies globally [9], and GDM prevalence is 14% [11], with up to 28% in some pregnant populations [11]. Pregnancy is therefore a unique opportunity to detect and manage risk factors for the development of future maternal CVD [12].

Altered lipid physiology and lipid accumulation will naturally occur with pregnancy progression, in response to physiological adaptations to foetal demands. However, evidence has shown that dyslipidaemia can predict pregnancy complications and adverse health outcomes [13, 14]. Specifically, dyslipidaemia has been linked extensively to the development of CVD [6, 15]. However, assessment of traditional lipid biomarkers including total triglycerides (TGs) and cholesterol does not reflect the complex lipid metabolism which occurs during pregnancy and in response to pregnancy-specific complications such as GDM [16]. Conversely, human lipidomic analysis has emerged as an in-depth and specialised approach to measuring a wide spectrum of lipid species [17]. Since its inception, lipidomic techniques have vastly expanded our understanding of the complexity of lipid dysregulation in cardiometabolic disease [17]. Lipidomic profiling during pregnancy may offer a novel tool for enhancing our understanding of altered lipid metabolism and identification of women who may require early pregnancy lifestyle intervention, such as healthy dietary modification, known to be associated with favourable lipid profiles [18] and lower risk of cardiometabolic disease [19–21].

Emerging research has shown that adherence to healthy dietary patterns including the Healthy Eating Index, Mediterranean Diet and the Dietary Approaches to Stop Hypertension (DASH) diet is associated with improved maternal health and reduced risk of pregnancy complications including GDM and gestational hypertension [19, 22]. However, data from mostly prospective cohort studies have shown that adherence to a single, preferred dietary pattern for decreasing risk of adverse pregnancy outcomes is yet to be determined. Further, assessment of diet quality (DQ) defined by adherence to national dietary guidelines and the impact on maternal lipidomic profiling has seldom been explored. The purpose of this study was to assess the association of early pregnancy DQ with maternal plasma lipidomic profiles at early and mid-pregnancy, blood pressure (BP) throughout pregnancy, diagnosis of GDM and total GWG.

## Materials and methods

### Study population

This study of pregnant women utilises data and biological samples collected as part of the Creatine in Pregnancy Outcomes (CPO) study. A detailed outline of the CPO study protocol and methodology has been previously reported [23]. Ethical approval for the original CPO study was granted in August 2015 from Monash Health (Ref 14140B) and Monash University (Ref 7785). The current study was granted ethical approval in July 2020 by the Deakin University Human Research Ethics Committee (DUHREC) (2020-236). Study procedures followed were in accordance with ethical standards of Monash Health, Monash University and Deakin University’s committees on human experimentation and conformed to the Declaration of Helsinki.

Women attending low-risk antenatal clinics at Monash Health in Melbourne, Australia, were recruited at 10–20 weeks gestation. Monash Health is a large tertiary teaching hospital in metropolitan Melbourne, Australia, and provides antenatal care to over 9000 women per year. Women gave informed, written consent to participate and agreed to have samples collected for the CPO study and biobanked for future research approved by Monash Health. For inclusion, women needed to be aged 18–40 years and classified as having a ‘low risk’, singleton pregnancy, meaning they had no known pre-existing medical or obstetric condition. Women were excluded if they were non-English speaking, had been previously diagnosed with Type 1/ Type 2 diabetes, taking creatine supplements during pregnancy, required high-risk pregnancy care due to underlying or emergent health complications or disclosed ongoing alcohol or drug use during pregnancy. The original CPO study was conducted between 2015 and 2017 and collected data for 284 women.

The current study utilised data from three antenatal visits; the first ([mean ± SD]) (15 ± 3 weeks gestation), third (27 ± 3 weeks gestation) and fifth (35 ± 2 weeks gestation) visits. We defined these timepoints as *early, mid-* and *late* pregnancy, respectively, consistent with common terms used to describe pregnancy progression [24, 25]. Demographic data included maternal age, education, gravida, smoking status, sex of the baby and gestational weeks at delivery. Region of birth was defined using birth countries combined into the following categories: Australian/New Zealand (Australia and New Zealand); Asian/South Asian (India, Nepal, China, Afghanistan, Singapore, Sri Lanka, Philippines, Malaysia, Pakistan, Bangladesh, Thailand); European/UK/Canada (Russia, Switzerland, Ireland, UK, Canada, Greece, France, Scotland, Poland, Germany, England); Middle Eastern/African/South American (Lebanon, Israel, Kuwait, Iran, Venezuela, Mauritius, Ghana, Ethiopia, Colombia).

### Dietary intake

Dietary intake was assessed using the Cancer Council of Victoria’s (CCV) validated Dietary Questionnaire for Epidemiological Studies (DQES version 2) [26]. Design of the DQES has been described in detail elsewhere [27]. This consisted of 74 items that assessed the frequency of intake across multiple food groups. The DQES is designed to reflect usual intake over the past 12 months. It has been previously validated against 7 day food diaries among reproductive age women [26], showing comparable performance to other widely used food frequency questionnaires [28] and has been used frequently as a validated tool for assessing dietary intake among Australian women [26] and pregnant populations [29, 30]. Women completed the online DQES at early pregnancy ([mean ± SD]) (15 ± 3 weeks gestation) and again at late pregnancy (35 ± 2 weeks gestation). Completion of the DQES at early pregnancy reflected dietary intake during the first trimester of pregnancy and approximately six months leading up to pregnancy. Dietary assessment via the DQES at late pregnancy assessed women’s dietary intake across all of pregnancy specifically. Intake responses from the 74 items are grouped into categories (cereal foods, sweets and snacks, dairy products, meats and fish, fruit and vegetables) and converted to a Dietary Guideline Index (DGI) score, indicating adherence to the Australian Dietary Guidelines (2013) for adults [31].

### Diet quality

The DGI has previously been used to assess DQ and risk of cardiometabolic disease in Australian adults [20, 32, 33]. The DGI included 11 food components (Table 1): diet variety; vegetables; fruit; grains and cereals; meat and alternatives; dairy and alternatives; discretionary foods; saturated fat; unsaturated fats; sugar and alcohol. Two additional components, fluid intake and limiting foods high in salt, were not included as the DQES version 2 did not include questions appropriate to score for those items. The DGI at late pregnancy was calculated to adhere to pregnancy specific guidelines which differ for the grains and cereals food group and meat and alternative food group compared to non-pregnancy guidelines. Possible scores for each food category were between 0 and 10 for nine components and 0 and 5 for two components, with a higher score in each category indicating better DQ and higher compliance with the Dietary Guidelines. The total DGI score for each participant ranged between 0 and 110, with a higher score indicating higher adherence to the Dietary Guidelines and, therefore better DQ [20, 32].

**Table 1:** Components of the Dietary Guideline Index and scoring criteria

Dietary guideline index indicator and description	Criteria^a^ for maximum score (10)		Criteria for minimum score (0)
	Early pregnancy^b^	Late pregnancy^c^	
1. Food variety: proportion of food from each core food groups eaten at least once per week	100%	100%	0%
2. Total vegetables intake: serves per day	5	5	0
3. Total fruit intake: serves per day	2	2	0
4a. Total cereal intake: serves per day	6	8.5	0
4b. Proportion of wholegrain to total cereals	> 50%	> 50%	0%
5a. Total meat and alternative: serves per day	2.5	3.5	0
5b. Proportion of lean meats and alternatives to total meats and alternatives	100%	100%	0%
6. Total dairy and alternatives: serves per day	2.5	2.5	0
7. Limit discretionary foods and drinks: serves per day	≤ 2.5	≤ 2.5	> 2.5
8. Limit saturated fats: proportion of reduced fat dairy and alternatives to total dairy and alternatives	> 50%	> 50%	0%
9. Small amounts of unsaturated oils, fats or spreads: serves per day	≤ 2	≤ 2	> 2
10. Limit added sugars: serves per day	≤ 1.25	≤ 1.25	> 1.25
11. Limit alcohol: serves per day	≤ 2	0	Early pregnancy (> 2)
			Late pregnancy (any)

^a^Based on recommendations of the Australian Dietary Guidelines (values represent serves unless otherwise stated)

^b^Early pregnancy (15 ± 3 weeks gestation, 12-month retrospective dietary intake in early pregnancy and nine months pre-pregnancy)

^c^Late pregnancy (27 ± 3 weeks gestation, retrospective FFQ reflects dietary intake during each trimester of pregnancy)

### Lipidomics

Comprehensive lipid profiling was undertaken, and 698 species across 37 lipid classes were measured. Such extensive lipidomic analysis allows for identification of important biomarkers for assessment of disease risk [34] and enables an understanding of lipid metabolism in pregnancy which would otherwise go undetected. Plasma lipids were analysed in samples collected at early ([mean ± SD) (15 ± 3 weeks) and mid (27 ± 3 weeks)-pregnancy for the current study. Blood was collected in lithium heparin tubes, stored on ice and spun (400 g, 20 min, 4°) to isolate plasma. Lipids were isolated from plasma using lipidomics analysis [35]. In brief, 10 μL of plasma was mixed with 90 μL of butanol/methanol (1:1) and 10 µL of an internal standard mix [35]. Samples were sonicated on a sonicator bath for 1 h, maintained at 25 °C and centrifuged at 13,000*g* for 10 min and the supernatant transferred into glass vials with inserts for mass spectrometry analysis. Lipidomic analysis was performed using high-performance liquid chromatography in conjunction with an Agilent 6490 QQQ mass spectrometer. Liquid chromatography was done using a Zorbax Eclipse Plus C18 (Agilent Technologies, USA), 1.8 μm, 100 × 2.1 mm column with running solvents comprising of water/acetonitrile/isopropanol at ratios of 50:30:20 and 1:9:90, respectively (A and B), both with 10 mM ammonium formate. The column was maintained at 45 °C during the run and the autosampler controlled at 25 °C. Lipid extracts (1 μL) were injected and separated under a stepped gradient condition with a flow rate of 400 μL/min as previously described [36]. An additional passivation step was performed prior to the run, where 0.5% phosphoric acid in 9:1 acetonitrile/water was run through the HPLC system for an hour and subsequently washed with 1:9 acetonitrile/water overnight. Additional characterisation of phospholipid structures was done as reported previously [36]. Each lipid was integrated manually via Agilent software. Normalisation was done between batches (3) using the pooled plasma QC’s spaced 20 samples in between each sample. This was conducted by median centering, where the median concentration of the QC’s for each batch was used to align the concentrations between each batch. Data from the lipidomic analysis were integrated using MassHunter V8.00 (Agilent, Australia). Relative lipid concentrations were calculated by relating the area under the chromatogram for each lipid species to the corresponding internal standard.

### Secondary outcome measures

Systolic and diastolic BP (mm Hg) was measured at each antenatal clinic visit by a research midwife, using Welch Allyn sphygmomanometers with manual cuff inflation and calibrated regularly as per manufacturers instruction. One reading was taken, and if abnormal, three consecutive readings were done at short intervals over 1.5 h, and the average of these readings was recorded. Medical history data (pre-existing medical conditions and GDM diagnosis) were recorded from hospital records. As part of routine care, all women without existing GDM undergo a 75 g oral glucose tolerance test at 24–28 weeks gestation [37]. A diagnosis of GDM was based on the International Association of the Diabetes and Pregnancy study groups (IADPSG) criteria; one of fasting glucose ≥ 5.1 mmol/L; 1 h level ≥ 10 mmol/L; or 2 h level ≥ 8.5 mmol/L [37]. Women’s height (cm) at the first antenatal clinic visit and weight (kg) using calibrated industrial scales at each clinic visit were measured by the same research midwife. Gestational weight gain (GWG) was calculated as weight at the last (fifth) clinic visit minus weight at the first antenatal clinic visit. Body mass index (BMI) (kg/m^2^) was calculated using World Health Organisation (WHO) BMI criteria for healthy weight, overweight or obese classifications [38].

### Statistical analyses

Data were analysed using STATA/SE statistical software version 16.0. Descriptive analyses reported maternal demographics, systolic and diastolic BP, GDM diagnosis and maternal anthropometry as mean ± SD or *n* (%). Dietary Guideline Index scores were reported as mean ± SD and the score range. Change in DGI scores from early to late pregnancy was assessed via paired *t*-tests for women who had complete FFQ data at both early and late pregnancy (*n* = 203). Paired-samples *t*-tests were conducted to assess mean changes in all lipids between early and mid-pregnancy. Linear and logistic regression models were used to assess associations between total DGI score at early pregnancy with continuous (plasma lipids, BP and GWG) and categorical (GDM diagnosis) outcomes, respectively. Predetermined factors known widely to influence lipids including age [39], BMI [40, 41] and gestational week [42, 43] were included as covariates in the regression models. The model examining the association between early pregnancy diet and mid-pregnancy lipid concentrations also included early pregnancy lipid concentrations as a covariate. Additional covariates included well established factors known to impact (i) BP in pregnancy (smoking [44], age [19, 45], early pregnancy BMI [19, 45] and gestational week at BP measurement [46]); (ii) GDM diagnosis (age [19, 47] early pregnancy BMI [48], country of birth [47]) and (iii) excess GWG ((age [49], early pregnancy BMI [50] and education [49]). These were further included in models assessing association of DGI with pregnancy complications. Finally, the Benjamini–Hochberg method [51] was applied to regression models involving lipid outcomes at both early and mid-pregnancy. This method is widely used for controlling the false discovery rate (FDR) using sequential modified Bonferroni correction for multiple comparisons [51, 52] and has previously been applied to regression models involving the plasma lipidome [36, 39]. A cut-off of FDR of 0.05 and a less conservative level for comparison of 0.2 were used for significance. In all other models (unadjusted and adjusted), statistical significance was set as *p* < 0.05.

## Results

### Participants

Of the 286 women enrolled, four women were excluded due to complete missing data, 52 women were excluded due to missing FFQ and anthropometric data and 15 women were excluded as their GDM status was unknown and not available to be retrieved from the electronic medical record system. This left data for 215 included the analyses (Supplemental material). Women were 31.5 ± 3.9 years ([mean ± SD]), and 64% of women were tertiary educated (Table 2). Just over half (54.9%) of all women were born in Australia/New Zealand, over one third (35.8%) were born in Asia/South Asia/Middle East/Africa/South America and 9.3% were born in Europe/UK/Canada. Over half of all women (56.3%) were pregnant with their first baby, and mean gestational age at delivery was 39.2 ± 1.4 weeks. Mean early pregnancy BMI was 25.0 ± 4.6 kg/m^2^. Women gained 10.9 ± 4.6 kg during pregnancy, and 9.7% of women were diagnosed with GDM. Mean blood pressure measures across pregnancy (systolic/diastolic) were 110/67 ± 12/9.3 mm Hg, 111/67.6 ± 11.0/7.8 mm Hg and 114./70.2 ± 12.1/9.8 mm Hg at early, mid- and late pregnancy, respectively, with a highest overall recorded measure of 122/77.1 ± 1.8/8.7 mm Hg.

**Table 2:** Characteristics of study participants^a^

	Mean ± SD or *n* (%)
Maternal age (years)	31.5 ± 3.9
Highest education (*n* = 214)	
Sub-tertiary	77 (36.0%)
Tertiary	137 (64.0%)
Region of birth	
Australia/New Zealand	118 (54.9%)
Asia/South Asia	65 (30.2%)
Europe/UK/Canada	20 (9.3%)
Middle East/Africa/South America	12 (5.6%)
Smoking status	
Non smoker	207 (96.3%)
Smoker	8 (3.7%)
Sex of baby (*n* = 213)	
Male	105 (49.5%)
Female	107 (50.5%)
SGA	16 (7.2%)
Preterm	8 (3.6%)
Gravida	
Primi	94 (43.7%)
Multi	121 (56.3%)
Gestation at delivery (weeks) (*n* = 211)	39.2 ± 1.4
BMI early pregnancy (kg/m^2^)	25.0 ± 4.6
BMI category early pregnancy	
Underweight	3 (1.4%)
Healthy weight	126 (58.6%)
Overweight	55 (25.6%)
Obese	31 (14.4%)
BMI late pregnancy (kg/m^2^) (*n* = 214)	29.1 ± 4.8
Gestational weight gain (kg)	10.9 ± 4.6
Gestational diabetes diagnosis	21 (9.7%)
Blood pressure early pregnancy (mm Hg) (*n* = 212)	
Systolic	110 ± 12.3
Diastolic	67 ± 9.3
Blood pressure mid-pregnancy (mm Hg) (*n* = 207)	
Systolic	111 ± 11
Diastolic	67 ± 7.8
Blood pressure late pregnancy (mm Hg) (*n* = 191)	
Systolic	114 ± 12.13
Diastolic	70 ± 9.8
Highest recorded blood pressure (mm Hg) (*n* = 210)	
Systolic	122 ± 10.8
Diastolic	77 ± 8.7

*BMI* Body Mass Index; *SGA* small for gestational age; < 10th centile

^a^*n* = 215 unless otherwise stated

### Diet quality

Compared to early pregnancy total DGI score ([mean ± SD]) (70.7 ± 11.4), DGI score at late pregnancy was statistically significantly lower (66.5 ± 12.6) (*p* < 0.0005) (Table 3). Scores for total cereal decreased significantly from early to late pregnancy (3.3 ± 1.2 and 2.5 ± 1.1) (*p* < 0.0005) and scores for total meat and alternatives also decreased significantly from early to late pregnancy (4.5 ± 1.1 and 3.9 ± 1.4) (*p* < 0.0005). Scores for total dairy and alternatives increased significantly from early to late pregnancy (6.3 ± 2.5 and 7.1 ± 2.5) (*p* < 0.0005). Intakes were poor for multiple key individual food components. Scores for limiting discretionary food items were well below the maximum score of 10 at both early and late pregnancy (4.0 ± 4.9 and 4.0 ± 4.9, respectively). DGI scores for vegetables and fruit (scored out of 10) were suboptimal at early and late pregnancy (3.3 ± 1.9 and 3.1 ± 1.8) (*p* = 0.078) and (7.2 ± 2.8 and 7.5 ± 2.8) (*p* = 0.128), respectively, where overall scores decreased for vegetables, although this difference was not significant. Further, DGI scores for limiting saturated fats and sugars were suboptimal decreased significantly from early to late pregnancy (3.6 ± 4.5 to 3.0 ± 4.4) (*p* = 0.018) and (5.1 ± 5.0 to 3.9 ± 4.9) (*p* ≤ 0.003), respectively.

**Table 3:** Summary of Dietary Guideline Index scores and change in scores^c^ at early and late pregnancy

Dietary Guideline Index indicator and description	Possible scores (min/max)	FFQ early pregnancy^a^ (*n* = 203)		FFQ late pregnancy^b^ (*n* = 203)		*p* Value
		Mean (SD)	Range	Mean (SD)	Range	
1. Food variety: proportion of food from each core food groups eaten at least once per week	0/10	5.4 (1.5)	0.2–9.2	5.3 (1.3)	1.5–8.5	0.168
2. Total vegetables intake: serves per day	0/10	3.3 (1.9)	0.3–10.0	3.1 (1.8)	0.2–9.8	0.078
3. Total fruit intake: serves per day	0/10	7.2 (2.8)	0.8–10.0	7.5 (2.8)	1.0–10.0	0.128
4a. Total cereal intake: serves per day	0/5	3.3 (1.2)	0.5–5.0	2.5 (1.1)	0.0–5.0	** < 0.0005**
4b. Proportion of wholegrain to total cereals	0/5	3.7 (1.8)	0.0–5.0	3.9 (1.7)	0.0–5.0	0.084
5a. Total meat and alternatives: serves per day	0/5	4.5 (1.1)	0.5–5.0	3.9 (1.4)	0.1–5.0	** < 0.0005**
5b. Proportion of lean meats and alternatives to total meats and alternatives	0/5	4.5 (0.5)	2.9–5.0	4.6 (0.4)	2.8–5.0	0.138
6. Total dairy and alternatives: serves per day	0/10	6.3 (2.5)	0.0–10.0	7.1 (2.5)	0.0–10.0	** < 0.0005**
7. Limit discretionary foods and drinks: serves per day	0/10	4.0 (4.9)	0.0–10.0	4.0 (4.9)	0.0–10.0	0.764
8. Limit saturated fats: proportion of reduced fat dairy and alternatives to total dairy and alternatives	0/10	3.6 (4.5)	0.0–10.0	3.0 (4.4)	0.0–10.0	**0.018**
9. Small amounts of unsaturated oils, fats or spreads: serves per day	0/10	10.0 (0.0)	10.0–10.0	10.0 (0.7)	0.0–10.0	0.319
10. Limit added sugars: serves per day	0/10	5.1 (5.0)	0.0–10.0	3.9 (4.9)	0.0–10.0	**0.003**
11. Limit alcohol: serves per day	0/10	10.0 (0.7)	0.0–10.0	7.7 (4.2)	0.0–10.0	** < 0.0005**
Total Dietary Guideline Index score	0/110	**70.7 (11.4**)	38.1–99.6	**66.5 (12.6)**	30.9–98.2	** < 0.0005**

^a^Early pregnancy (15 ± 3 weeks gestation) 12-month retrospective dietary intake in early pregnancy and nine months pre-pregnancy

^b^Late pregnancy (35 ± 2 weeks gestation) reflects dietary intake during each trimester of pregnancy

^c^Change in Dietary Guideline Index score assessed by paired *t*-tests; FFQ (Food Frequency Questionnaire)

### Plasma lipids

**Fig. 1:**
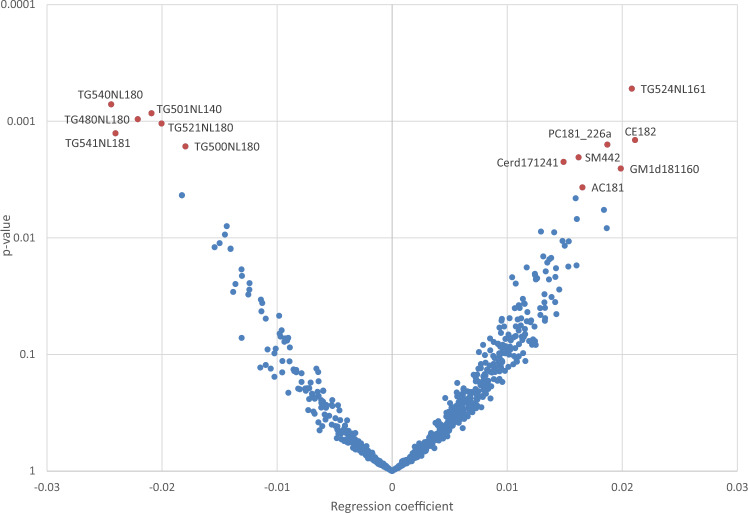
Associations of early pregnancy diet quality (diet quality assessed by total dietary guideline index score; the model included the Benjamini–Hochberg procedure applied at a FDR of 20%; linear regression adjusted for early pregnancy plasma lipid levels, maternal age, BMI and gestational week) with mid-pregnancy plasma lipids

Changes in lipid level were observed for the majority of the lipids, with most increasing significantly from early to mid-pregnancy, while a smaller proportion decreased significantly from early to mid-pregnancy (Supplemental material). There were no statistically significant associations found between DGI score at early pregnancy and lipid outcomes at either early or mid-pregnancy when the model was adjusted for a FDR of 0.05 using the Benjamini–Hochberg procedure (Supplemental material). When we applied a FDR of 0.2, there were multiple statistically significant associations between early pregnancy DGI score and plasma lipids at mid-pregnancy, but not early pregnancy. We run an unadjusted model for all lipids and found that only one lipid (LPCO221) reached significance at a FDR of 0.2, despite not being significant in the adjusted model (data not presented). There was no significant association found in unadjusted models at a FDR of 0.05. Figure 1 presents associations between DGI score at early pregnancy with 698 lipid species at mid-pregnancy. Under the FDR of 0.2, significant associations were found for 13 of the 698 lipids. These significant associations are further summarised in Table 4. Total DGI score at early pregnancy was significantly associated with seven plasma TGs (out of 113 total TG species) at mid-pregnancy, six of which were negative associations. Three TGs contained the 18:0 saturated fatty acid stearic acid ((TG54:0)[NL18:0]; (TG48:0)[NL18:0]); (TG50:0)[NL18:0]). Total DGI score was significantly positively associated with one unsaturated fatty acid ((TG52:4)[NL16:1]), palmitoleic acid.

**Table 4:** Significant associations between diet quality^a^ at early pregnancy and plasma lipids at mid-pregnancy^b^

Lipid species	β (95% CI)	*p* Value
**TG524NL161**	0.02 (0.11, 0.03)	0.0001
**TG540NL180**	− 0.02 (− 0.04, − 0.01)	0.0001
**TG501NL140**	− 0.02 (− 0.03, − 0.01)	0.0001
**TG480NL180**	− 0.02 (− 0.04, − 0.01)	0.0001
**TG521NL180**	− 0.02 (− 0.03, − 0.01)	0.0010
CE182	0.02 (0.01, 0.03)	0.0015
PC181_226a	0.02 (0.01, 0.03)	0.0016
**TG541NL181**	− 0.02 (− 0.04, − 0.01)	0.0013
GM1d181160	0.02 (0.01, 0.03)	0.0026
**TG500NL180**	− 0.02 (− 0.03, − 0.01)	0.0016
SM442	0.02 (0.01, 0.03)	0.0020
Cerd171241	0.01 (0.01, 0.02)	0.0023
AC181	0.01 (0.01, 0.03)	0.0037

^a^Diet quality assessed by total dietary guideline index score

^b^Multiple linear regression adjusted for 0.2 false discovery rate (Benjamini–Hochberg procedure) and early pregnancy BMI, maternal age, gestational week and plasma lipids at early pregnancy

### Blood pressure, gestational diabetes and gestational weight gain

A statistically significant inverse association between DGI score and diastolic BP at late pregnancy was found (−0.14 mm Hg, 95% CI −0.26 −0.02, *p* = 0.028) (Table 5). No statistically significant associations were found between DGI score at early pregnancy with BP at any time point, GDM diagnosis (26–28 weeks gestation) or total GWG.

**Table 5:** Associations of early pregnancy diet quality^a^ with blood pressure, gestational diabetes and gestational weight gain

Early pregnancy Dietary Guideline Index score	β (95% CI)	OR (95% CI)	*p* Value
Gestational weight gain^b^ (kg) (*n* = 213)	0.05 (− 0.00, 0.11)		0.069
Gestational diabetes mellitus^c^ (*n* = 211)		0.99 (0.95, 1.03)	0.629

Early pregnancy Dietary Guideline Index score	Blood pressure^d^ (mm Hg)	β (95% CI)	*p* Value
	Early pregnancy blood pressure		
	Systolic	0.01 (− 0.14, 0.15)	0.938
	Diastolic	0.03 (− 0.08, 0.14)	0.583
	Mid-pregnancy blood pressure		
	Systolic	0.08 (− 0.05, 0.22)	0.224
	Diastolic	0.07 (− 0.02, 0.16)	0.107
	Late pregnancy blood pressure		
	Systolic	− 0.02 (− 0.17, 0.12)	0.742
	Diastolic	− 0.14 (− 0.26, − 0.02)	**0.028**

*n* = 215 unless otherwise stated

^a^Diet quality assessed by total dietary guideline index score

^b^Gestational weight gain assessed as difference between late pregnancy weight and early pregnancy weight, linear regression adjusted for maternal age, early pregnancy BMI and education

^c^Gestational diabetes mellitus diagnosis (24–28 weeks gestation), logistic regression adjusted for early pregnancy BMI, country of birth and maternal age

^d^Blood pressure assessed at early, mid- and late pregnancy, linear regression adjusted for smoking, early pregnancy BMI and gestational week at blood pressure measure

## Discussion

To our knowledge, this study was the first to explore the relationship between the DGI and maternal lipids, utilising lipidomics. Our findings provide novel insights into the potential impact of early pregnancy diet on important lipid biomarkers. We also provide a unique assessment of DQ across pregnancy, and its association with important pregnancy outcomes as markers of maternal cardiometabolic health. Pregnancy has been regarded a ‘missed opportunity’ for CVD prevention [53]. The novel and comprehensive application of lipidomic analysis in our study has shown it is possible to identify abnormalities across an extensive range of plasma lipids in pregnancy, which would otherwise go undetected.

The relationship between dietary guideline adherence and maternal lipid profile was strongest for the TG lipid species when the model was adjusted for a FDR of 0.2. A higher total DGI score at early pregnancy was associated with lower levels of six TGs at mid-pregnancy (27 ± 3 weeks gestation), all containing saturated and monounsaturated long-chain fatty acid tails. Specific NL observations with these lipids highlighted observing fatty acid 14:0, 18:0 and 18:1 within these TG headgroups. Whilst past research has shown that saturated fatty acids containing 12–16 carbon atoms have the greatest effect on LDL cholesterol concentration and subsequent CVD risk [54], stearic acid has been shown to be a major contributor to development of ischemic heart disease [54, 55], inflammation and lipotoxicity [54]. Further, a significant, positive association was found between total DGI score and one monounsaturated TG, palmitoleic acid (16:1). The link between palmitoleic acid and cardiometabolic health is less clear. However, epidemiological studies have linked palmitoleic acid to cholesterol metabolism, increased insulin sensitivity and glucose tolerance [56]. High dietary intakes of palmitoleic acid have also been shown to be associated with lower blood LDL cholesterol concentrations [54, 57]. Our findings therefore suggest that healthier dietary intakes during pregnancy may have a potential role in modifying TG levels during a critical period when women are vulnerable to dyslipidaemia. Whilst further work is needed to understand the complex relationships between early pregnancy diet and TG levels, in the context of pregnancy-related cardiometabolic health, our findings have revealed the usefulness of utilising lipidomic assessment in examining this relationship.

The importance of dietary intakes which adhere to dietary guidelines during pregnancy extends beyond the need to support optimal growth and development of the foetus, but importantly, to positively impact maternal health. Recent evidence has shown that maternal dyslipidaemia is linked with multiple, adverse maternal and foetal outcomes [53, 58, 59]. Specifically in relation to TGs, elevated saturated/low unsaturated levels have been shown to predict GDM in the few studies that have used lipidomics to quantify lipid profiles in pregnancy [16, 60]. The impact of dyslipidaemia on maternal health can also persist far beyond pregnancy. For example, data from the Generation R study, (*n* = 5690) assessed early pregnancy maternal lipid profiles at 13 weeks gestation and found that TG and remnant cholesterol levels in early pregnancy were associated with long-term postpartum hypertension at 6 and 9 years [13]. Therefore, pregnancy is a unique life-stage which offers an opportunity to identify and appropriately manage, risk factors for current and future cardiometabolic disease in the clinical setting.

We found that DQ scores reflected low adherence to the Australian Dietary Guidelines, findings similar to other studies which have assessed dietary intake during pregnancy [61–63] and across non-pregnant populations [64]. In our study, intake of fruit and vegetables and discretionary food items did not adhere to guidelines. Whilst many barriers to implementation of dietary guidelines during pregnancy have been identified, including pregnancy symptoms such as fatigue and nausea [63, 65] inadequate provision of nutrition counselling from antenatal healthcare providers [65, 66] and a lack of knowledge regarding healthy eating during pregnancy [61, 67], adherence to dietary guidelines assessed via DQ indices has been consistently shown to reduce the risk of cardiometabolic disease [32, 68]. This indicates the overall potential long-term benefit of achieving dietary intakes which adhere to dietary guidelines and underpins pregnancy a critical period. Pregnancy care practices should ensure women are adequately supported to achieve optimal dietary intakes for both short- and long-term health benefit.

Aside from an association between DQ and diastolic BP at late pregnancy, we found no other significant associations between DQ and BP or GDM. Previous studies have shown a reduced risk of GDM with dietary intakes high in fruit and vegetables, whole grains and legumes, yet overall, findings have been mixed [69]. In the only systematic review and meta-analysis to date to have evaluated the impact of DQ using defined adherence to dietary guidelines during preconception and pregnancy on adverse perinatal outcomes [22], pooled data from 33 prospective cohort studies showed that higher DQ was associated with lower risk for GDM and preeclampsia [22]. Specifically, the Mediterranean Diet and the Dietary Approaches to Stop Hypertension (DASH) diets have shown promising results in reducing risk for pregnancy complications, including gestational hypertension. They have previously been associated with improved glucose and lipid metabolism and with lower systolic and diastolic BP [19, 70]. Variation in dietary approaches across DQ assessment (e.g. Mediterranean diet, Prime Diet Quality Score, the Alternative Healthy Eating Index and the Nordic Diet) points to the need for further large prospective cohort and population studies which assess DQ with pregnancy outcomes, to determine a preferred dietary pattern for use in early pregnancy interventions.

We found no association between DQ at early pregnancy and total GWG, a finding consistent with the literature to date reporting this relationship [22]. Perhaps not surprisingly, overall energy intake rather than DQ has been suggested to be the main driver of GWG during pregnancy [22, 71]. Excess GWG is a significant contributing factor to postpartum weight retention [72] and should be routinely monitored as part of screening for risk factors which impact women’s cardiometabolic health during pregnancy.

### Strengths and limitations

A major strength of our study was the utilisation of comprehensive human lipidomic analysis. This robust method has enabled insights into the potential impact of diet on lipid metabolism at the molecular level in pregnant women. This was also the first study to our knowledge, to have assessed the relationship between DQ and maternal plasma lipidomics during pregnancy, including measures across two trimesters. An additional strength of this study was using the Australian DGI to calculate DQ at both early and late pregnancy. Dietary intake assessment has shifted from evaluating individual nutrient intake to whole foods and food patterns [73] to account for important interactions of nutrients and non-nutrient components [73]. Therefore, a major strength of the DGI is that it is a validated, food-based index and is translatable to public health messages related to the whole diet [74]. Our study also has some limitations. Recruitment of a high proportion (64%) of tertiary educated women with low-risk pregnancies means that findings are not generalizable to all pregnant women, especially those at ‘high risk’ of maternal complications. Assessment of DQ in less educated populations would enable important identification of vulnerable pregnant women, as poorer DQ may be expected in women of lower socioeconomic status and lower education level. Future research might also recruit women with more complicated pregnancies or women who are at high risk for adverse pregnancy outcomes, specifically women with a pre-pregnancy BMI > 30 kg/m^2^. A further limitation was the use of self-reported dietary data which may be prone to recall bias or social desirability (e.g. overreported fruit and vegetable intake). However, as mean DGI scores of healthy foods including fruit and vegetables fell short of meeting dietary guidelines, this would suggest that women did not overreport intake of healthy foods. The DQES is commonly used to assess usual intake over the past 12 months and despite having been previously validated against weighed food records, it may not entirely reflect usual dietary intake due to recall of intake over a relatively long timeframe. Further, we did not account for women’s physical activity levels in our study, which is important when considering the effect on plasma TGs. We recognise that there is a need to consider the effect of lifestyle factors including physical activity in interpreting lipidomic profiles in pregnancy, as physical activity has shown positive effects on lowering fasting and postprandial blood glucose levels and blood triglyceride concentrations [75]. Finally, as this was a low-risk sample of pregnant women without serious complications, we could not assess associations of DQ with preeclampsia. However, including BP outcomes across pregnancy was an important aspect of our study as BP monitoring of levels below clinical thresholds of gestational hypertension or preeclampsia is still important in the assessment of overall CVD-related health.

## Conclusions

This study has offered novel insight into the impact of maternal diet on important lipid biomarkers in pregnant women. Further work which focuses on lipidomic analysis in early pregnancy to determine the best dietary approaches for targeting maternal lipid metabolism and cardiometabolic health in pregnancy is needed. Our analyses highlight the sensitivity of TGs to dietary intakes during pregnancy. Given the established relationships between TG metabolism and cardiometabolic health, our findings suggest that comprehensive analysis of TGs during pregnancy could be of benefit in early antenatal assessment. Future studies which assess maternal cardiometabolic health outcomes in relation to modifiable factors are needed.

### Supplementary Information

Below is the link to the electronic supplementary material.

Supplementary file1 (DOCX 33 KB)

Supplementary file2 (XLSX 420 KB)

Supplementary file3 (XLSX 126 KB)

## Data Availability

Data described in the manuscript, code book, and analytic code will be made available upon request pending reasonable application and approval.

## Abbreviations

BP: Blood pressure
GWG: Gestational weight gain
BMI: Body Mass Index
DGI: Dietary Guideline Index
GDM: Gestational diabetes mellitus
CVD: Cardiovascular disease
DQ: Diet quality
FFQ: Food frequency questionnaire
TG: Triglyceride
